# Disabled people in Britain and the impact of the COVID‐19 pandemic

**DOI:** 10.1111/spol.12758

**Published:** 2021-08-11

**Authors:** Tom Shakespeare, Nicholas Watson, Richard Brunner, Jane Cullingworth, Shaffa Hameed, Nathaniel Scherer, Charlotte Pearson, Veronika Reichenberger

**Affiliations:** ^1^ International Centre for Evidence in Disability LSHTM London UK; ^2^ Centre for Disability Research University of Glasgow Glasgow UK; ^3^ Urban Studies University of Glasgow Glasgow UK

**Keywords:** COVID‐19, disability, information, lockdown, pandemic, social care, social distancing

## Abstract

This paper reports on in‐depth qualitative interviews conducted with 69 disabled people in England and Scotland, and with 28 key informants from infrastructure organisations in the voluntary and statutory sectors, about the impact of COVID‐19, and measures taken to control it. Participants were recruited through voluntary organisations. As with everyone, the Pandemic has had a huge impact: we discuss the dislocations it has caused in everyday life; the failures of social care; the use of new technologies; and participants' view on leadership and communication. We conclude with suggestions for urgent short term and medium term responses, so that the United Kingdom and other countries can respond better to this and other pandemics, and build a more inclusive world.

## 1 INTRODUCTION

In the United Kingdom there are over 11 million disabled people ((Office for National Statistics, 2019a, 2019b). They account for roughly 20% of the country's population and at the start of the pandemic there was widespread concern about their susceptibility to SARS‐CoV‐2 virus and the resulting COVID‐19 diseases. Of those aged 65 and over, 45% are disabled (Office for National Statistics, 2019a, 2019b), and older people are known to be at greater risk of the virus (Harrison et al., 2020). Certain groups, such as those with organ transplants, those living with severe respiratory conditions such as cystic fibrosis or those who have specific cancers, such as blood or bone marrow were singled out as being at particular risk of increased morbidity or mortality associated with C0vid‐19 (Harrison et al., 2020; Yang et al., 2020). This group was later expanded to include other conditions such as people with a neurological condition, people post‐stroke and people with diabetes. There was a general concern that the narrower margin of health experienced by many disabled people (World Health Organization, 2011) would make them particularly at risk from COVID‐19.

Not only was it felt that disabled people were at increased risk of morbidity and mortality following infection, there was also concern that many disabled people were in living arrangements or receiving care and support services in ways which increased their vulnerability (Dickinson et al., 2020). For example, many disabled people live in congregate settings, placing them at increased risk of COVID‐19 transmission (Daly, 2020). Those disabled people who rely on domiciliary social care provided by support workers and personal assistants were also seen as being placed at risk, as many care workers visit large numbers of disabled people (Glynn et al., 2020). Self‐isolation for many disabled people is difficult or impossible. The United Kingdom's Coronavirus Act (2020) suspended the Care Act (2014) in England, and in Scotland the duty for Local Authorities to assess need, which raised fears that social support needs would not being met in every circumstance.

COVID‐19 placed considerable pressure on already overstretched services. Since the global financial crisis of 2008, and the election of Coalition/Conservative governments, cuts have affected resilience of disabled people (Mladenov, 2015). More targeted welfare benefits, sanctioning, and reductions in entitlements have affected those unable to work (Glasby et al., 2020). The Institute for Government estimate that between 2009/2010 and 2014/2015, local authorities in England cut spending on adult social care by nearly 9.3% in real terms and that by 2019 social care funding had been cut by 2% in real terms compared to 2008/2009 (Institute for Government, 2019). Cuts of this scale to an already over‐burdened service have meant reductions in the range and quality of services, eligibility criteria have been changed to reduce access, and there has been an increased reliance on informal care provided by family and friends (Glasby et al., 2020; Morris, 2011).

Despite the raised COVID‐19 risk—clinical and social—experienced by disabled people, this population is strangely missing from important analyses which have been published during the Pandemic. For example, Andrew et al. (2020) talk about inequalities in children's experiences of home learning during COVID‐19, but fail to mention children with special educational needs and disabilities. Hupkau and Petrongolo (2020) talk about care and gender, but fail to mention disability, disabled children or disabled parents. Public Health England (2020) talk about disparities in risk and outcomes of COVID‐19, and so does Bibby et al. (2020), but neither mention disability, despite discussion of economic, gender, age and racial disparities. The same goes for Johnson et al. (2021). It is astounding that disability is absent from these studies. It is complex but not hard to disaggregate disability. However, there may be difficulties bringing together evidence for which specific health conditions put people at higher risk of COVID‐19.

Early reports on the impact of the pandemic and the response to curtail its spread give substance to many fears from the disability community. Disabled people's organisations and activists have carried out surveys of their members and have drawn attention to the disproportionate impact of the COVID‐19 pandemic on the disability community (Campbell, 2020; Greater Manchester Coalition of Disabled People, 2020; Inclusion London, 2020; Inclusion Scotland, 2020). Glasgow Disability Alliance, for example, telephoned over 5,000 disabled people across Glasgow in the early months of the pandemic to survey their wellbeing. Their report concludes that the barriers disabled people face and the inequality they experience has made them less able to respond to the challenges COVID‐19 has placed on them. They also found that disabled people have been excluded from the decision‐making process and that their needs have been overlooked. A survey carried out by Inclusion London with over 300 respondents concludes that disabled people are ‘are experiencing increasing levels of psychological distress, social isolation, a lack of social care support, workplace discrimination, food poverty, and unequal access to health care’ (Glasgow Disability Alliance, 2020, 4).

There is now also a growing body of quantitative evidence to suggest that the changes implemented to prevent the spread of COVID‐19 and the request for people to ‘stay home to stay safe’ has had a considerable impact on the wellbeing of disabled people with many reporting increased mental health problems (Office for National Statistics, 2020b, 2020c; Theis et al., 2020). Above all, there is now good evidence that disabled people have died in disproportionate numbers during the pandemic (Office for National Statistics, 2020a). This is well known for older people (Sinnathamby et al., 2020), but is also particularly the case for people with intellectual disabilities (LeDeR, 2020).

However, there has to date been little detailed qualitative research exploring the impact of COVID‐19 on disabled people, their lives and the services they receive. This paper aims to fill that gap. Drawing on interviews with 69 disabled people and 28 disability organisations, it provides authoritative evidence about how disabled people in England and Scotland are experiencing the COVID‐19 pandemic. We describe how COVID‐19 has disrupted their lives and their support, the impact it has had on the provision of social care, the role of new technologies and messaging and leadership. Based on our analysis of this data, we recommend the response measures to help disabled people and their families, both now and as we emerge from this crises. This study has highlighted existing inequalities, and the need to bring back better after the pandemic.

## 2 METHODS

The data presented here are drawn from a UKRI‐funded study exploring the impact of the pandemic on disabled people. We adopted a strategy that would allow us to collect detailed evidence of the experiences of individual disabled people but also provide breadth to the study. In total, 69 in‐depth semi‐structured interviews were conducted with disabled people, including 11 carers of disabled children. People who were interviewed had a wide range of impairments: physical, sensory, intellectual and mental health conditions. People with dementia and autistic people were also included. We conducted 28 semi‐structured interviews with organisations of and for disabled people and other key informants including social services. These data are from the first wave of interviews in June–August 2020; we will be carrying out a second wave of interviews in February–April 2021. Full ethical review and approval has been conducted by the London School of Hygiene & Tropical Medicine Research Ethics Committee.

### 2.1 Participants

In total, 30 interviewees were recruited from England (mainly Greater London and East Anglia) and 38 from Scotland (mainly Glasgow but also across Scotland: 41 were female, 27 male, and one gender neutral, four identified as being from a Black or Minority Ethnic (BME) community. A total of 33 lived with other family, 26 lived alone, and seven lived in a residential setting. We sought to recruit people from urban, suburban, rural and remote‐rural settings. The breakdown by impairment category is given in Table 1, noting that many participants reported more than one impairment. Participants were recruited via a range of disabled people's organisations and other NGOs. All interviewees were volunteers and were given a £20 voucher to compensate for their time. We approached organisations and asked them to publicise the research to their members, leaving it up to individuals to contact us. Working with us, the organisations used a range of methods including publicising the research on their web sites, emails to their members and mailshots. We wanted to ensure that we recruited participants who did not have Internet access, and worked with organisations to specifically target this group. All participants were guaranteed anonymity and all names used are pseudonyms.

**TABLE 1:** Respondents by impairment category

Impairment	Number
Autism/neurodiversity	8
Cognitive impairment	5
Intellectual impairment	19
Mental health condition	18
Physical impairment	33
Sensory impairment	15
Total (some participants had multiple conditions)	98

Interviews were conducted remotely, via telephone, Zoom, or where requested, by email. The pandemic has meant that we have all had to adapt and change our way of working and there has been a huge increase in the use of video conferencing and other technologies as a means of data collection (Lobe et al., 2020) All three modes of interview have previously been shown to be useful: the quality of data obtained does not differ markedly from face to face interviews, although we found it can be harder to develop rapport with people with learning disabilities online (Deakin & Wakefield, 2013; Mason & Ide, 2014; Mealer & Jones, 2014). Our interviews took between 30 and 60 min. All the interviews were transcribed verbatim for subsequent analysis. Each interview was then coded using NVivo 12. Thematic analysis (Guest et al., 2012) provided the framework and involved initial coding of interview transcripts to identify the key themes emerging from the data. These were discussed across the entire team, and a coding scheme and codebook was developed collectively and iteratively. The team exchanged transcripts and cross‐reviewed coding of eight transcripts to maximise consistency of coding across the whole project.

## 3 RESULTS

The COVID‐19 pandemic has significantly impacted on disabled people and their lives. A CEO of a large disability organisation put this succinctly:

I mean without being dramatic I think it's been catastrophic. I think it has taken existing inequalities that disabled people experience and it has magnified them and exaggerated them. (SO12)

In this section we present the emerging key findings from our interviews under four themes. First, we explore how the pandemic has impacted on people's day to day lives and their social routines, showing the dislocation it has caused. Second, we examine the role of social care and how this has been affected by the pandemic. Third, we explore the ways in which new technologies have been used by disabled people. Finally, we examine participants' perspectives on leadership and messaging. These themes were both common and highly relevant in this crisis. It is urgent to resolve these problems and minimise these disadvantages.

### 3.1 Disabling disruptions

In this section, we document the way that the pandemic has disrupted the established social practices and routines of our research participants, impacting for many on their mental health and wellbeing. While this disruption will be a common feature of the pandemic for all, there are specific issues faced by disabled people that have magnified that disruption. Disabled people have a narrower margin of health (WHO, 2011) and many of our participants feared that their impairment or other co‐morbidity would place them at significant risk of harm, should they be infected by SARS‐CoV‐2. Many disabled people are more dependent than non‐disabled people on medical or rehabilitation interventions, and disruption to services therefore affects them more (Dickinson et al., 2020). All the organisations we spoke to expressed concern about the long‐term impact of COVID and the lockdown on disabled people's mental health and wellbeing.

People described how their health care and support had changed significantly. Routine physiotherapy, speech and language therapy and occupational therapy were cancelled, causing particular problems for young disabled people. Attempts to replicate these therapies either via vide0 conference or phone were not perceived to be particularly successful. Caregivers expressed real concerns of long‐term negative impacts for disabled children, affecting their health and development. Many routine annual check‐ups were cancelled, raising the risk of preventable medical problems being missed. Provision, repair and service of assistive products and aids to daily living was severely affected. All this may lead to lack of functioning and increased dependency, with potential negative impacts on caregivers. The young people with disabilities and their parents/guardians we spoke to commented on how they have lost up to a year of therapy, education and socialisation. Other groups of disabled people reported similar impacts on assistive technology, rehabilitation and other therapies. For example, people with dementia reported losing confidence about participating in the mainstream, a decline in their physical capacity and the ability to travel independently.

Having an impairment increased the impact of the pandemic for many (Theis et al., 2020). Lily, for example, who struggles with mental health issues and obsessive compulsive health anxiety, described the effect it was having on her:

I can't go out for the fear of dying, and then when I do go out, sorry, when I do go out, I'm running… I'm moving away from people quicker (S19).

The demands of social distancing interfered with people's ability to communicate and participate, so Alan told us:

I'm missing out on, say…you know, it's serious. You know, it's…like, missing out the shaking hands with people and given them a hug and things like that. As a blind person I'm missing out on the whole lot because of social distancing. (S15).

People have also talked about how they had faced increased stigma, particularly those whose impairment mean they have trouble maintaining social distance:

You know, it's the social distancing. LD, dementia, blind. Oh, yeah. Blind. Yeah. assistance. Dogs are not trained in social distancing. Don't jump the queue and go straight for the door. Can you imagine the social consequences of that? So I've got any number of blind friends with assistance dogs, who are normally really independent, who are now not going out except with family, because they're saying the risk of them bluntly being thumped is too high. (E21).

People who were deafened or hard of hearing talked about the problems facemasks caused and the abuse they received if they asked people to remove them to help understanding: transparent masks for lipreading have been in very short supply, leaving people excluded from the spoken world.

Connected to these problems is an even greater social and political problem, which is that disabled people appear to have been an afterthought in the response to COVID‐19. Perhaps because they are a minority—perhaps 15%–20% of the population—they have been neglected in responses which have prioritised the majority. For example, we heard how provision was often made for non‐disabled children who were learning from home, but not, at first, for children with special educational needs and disabilities, and learning materials were often inaccessible or inappropriate. As the CEO of a large organisation of disabled people told us:

And as usual social care and disabled people in the community were the Cinderella, even more so than the care homes. Care homes hit the headlines first. … Whereas we were always at the end of the list. We're even more so at the end of the list. Last thing people think about. …you know, people don't think about people trying to live their lives in the communities in the same way. (SO11).

Personal Protective Equipment (PPE) was provided for hospitals, but not for care homes, and then in care homes, but not for homecarers and personal assistants. Some disabled people's organisations had to step in and source PPE for their members. One of the organisations we spoke to were so successful at this that they became, for a while, a major centre for the distribution of PPE for the area, replacing the statutory health and social care providers. Symbolic of disabled people being an afterthought is that at the time of writing, the Prime Minister's regular 5 PM. television briefings have not been sign language interpreted for Deaf people (in contrast to the daily briefings in Scotland that were interpreted from day one). Ethan, who is Deaf, describes having to watch a news report and a British Sign Language (BSL) translation of it on a different channel. When asked whether he felt that needs of disabled people had been adequately considered by the government during this epidemic, he replied:

No, certainly not, certainly not, and definitely not BSL users. Lowest of the low, we're right at the bottom of their list and falling off of it. (E11).

The same is true for people who were deafened or hard of hearing but were not BSL users, with little concern or thought given to meeting their needs, despite their prevalence in the community.

### 3.2 Social care reversions

Our research participants described how the pandemic and protective measures to avert contagion had led to increased reliance on their family and other informal carers, There were two key drivers for this. First was the closure, or suspension of day centres, day services and large sections of the social care system, large numbers of social care contracts were cancelled, put on hold, or severely limited. Second, some of our participants were anxious about having too many people coming into their own homes and wanted to reduce contact. As a result, where it was possible, they preferred using family members who were already part of ‘their social bubble’. In many cases this was made easier because their partners, parents or other informal carer were furloughed or were working from home and were able to provide this support. Many of those we spoke to reported concern about the impact this may have on the security and stability of their care in the future.

If new needs arose it was often hard to get support and in some areas social care assessments were suspended for up to 4 months, leaving those with newly acquired impairments or where support needs increased, without the help they required:

I've … been stuck upstairs for fourteen weeks because my [stair]lift has broken down and the local authority has been arguing with me about replacing the lift. They're wanting me to live downstairs. I've stayed in my bathroom, my study and my bedroom after fourteen weeks. (S02 Jonathan, physical impairment).

Before COVID, Michael who has autism and intellectual disabilities, lived in his own house with support. He moved back in with his elderly parents because of the closure of support services and the family's fear of him contracting COVID. This affected both his physical and mental health as his sister Alice told us:

Then the next thing that happened was his day centre closed pretty rapidly so all that day care support that he had went overnight, so that was another rapid change for him… It really hit him really hard and his behaviours that had been well under control, sort of repetitive behaviours, behaviours that are distressing for him, lashing out verbally, lashing out physically, started to come back and we started to see quite a rapid breakdown in his mental health, basically. Also on top of that, he has life threatening epilepsy and… he had five seizures in the first few weeks of lockdown whereas he would normally have one every six weeks. We consulted his consultant on that and he said it was down to the stress of what was happening in his routine due to the COVID outbreak and that he was seeing that in many other patients as well. (S27 Michael, interview with Alice, sister and guardian).

Social services appear to have been largely absent in some authorities where we conducted interviews:

They're just not answering their phones. Social work and that, you're not allowed to have their email address so you're literally…you've got to just sit and hope for the best that they phone you, as simple as that. There's only so much anybody can take.[…] People don't realise like how much harder it is for people that are severely isolated, mental health or disabilities it's been a nightmare, it really has. (Hannah, S21, physical impairment/mental health issues).

People told us how for some funding for their normal support services had been stopped completely and they had been left without any other alternative. Others had been offered phone support, one person we spoke to for example described how his support had been reduced from 12 h a week to one short phone call a week. A mother of a young man with profound learning disabilities described how the normal respite and short break support she received had been stopped completely and she had not been contacted by social care for over 4months.

Coupled with this was a new uncertainty about the future funding of social care—particularly given the vast increase in public sector borrowing. Many of the organisations we spoke to expressed fears about a reversion to a residualist state with responsibility placed in the family, as the ‘carer of last resort’, where aspirations to participation and independent living become a thing of the past for many people. Many families are struggling, both financially and emotionally. And for many, it has caused deep stress, when juggling caregiving and working from home, and for parents of young children, schooling also thrown in the mix. Lockdown and furlough has at least meant that in some cases thay are able to provide this support, but this will cease when there is a return to normal patterns of work.

Another dimension of this has been how the pandemic has illuminated the fragility of social bonds for disabled people, particularly people with intellectual disabilities. Many daily activities are not under their control. The valuable communities and bonds they create are fragmented and piecemeal, contingent on the support of others. If a person goes to a day centre, or a drama group, or goes shopping, this is activity depends on availability both of state funding, and of support workers. Once funding and staffing are withdrawn, a person with autism or intellectual disability or a mental health condition might be isolated, spending most of the day alone or inside, and with no meaningful activity. There is increased anxiety and loss of confidence. For example, Basil, who has intellectual disability and lives together with his partner, said:

On a Tuesday, I go to life skills before the lockdown but since the lockdown I haven't been able to go 'cause they wouldn't leave, and all packed up 'cause of the virus. I'm wanting to get back but we can't 'cause it's the problems with the buses and everything (E12).

Archie, a young man with cystic fibrosis, autism and an intellectual disability, has lost many of his day to day activities, as his caregiver explained:

So he belonged to swimming clubs and disability football clubs, disability basketball clubs, so he's day involved around seeing lots of people. And it's, you know, the other people within his peer group. And that has completely stopped. Before all of this he very, very rarely did anything with us as a family. Because he found it too difficult and so everything he did was one on one. If he did do something with us it would be one on one rather than a family event. So, we didn't really see a lot of him, because he was always out with his PA, and that's been in contrast to how it's been now that's been quite different. But he doesn't really know how to, he isn't very good in group situations, he is very good one‐on‐one, but in that we have six of us in our household so that is a group. And so he spent the majority of the time confined to his bedroom, watching TV, which is his favourite thing to do and he's very happy doing that… is a very different life to the one that he knew before. (Vanessa, E14).

For many we spoke to, with limited social options, boredom was one of the key features of the lockdown period:

There's nothing I can do to make my day shorter. At the moment I'm trying to sleep to get rid of some of the day. (Megan, S10).

These issues would have been much worse but for the role played by the third sector. At the start of the pandemic many organisations completely changed the way they work, filling in the gaps left by social care and making a material difference to people's mental health and wellbeing:

ENABLE have been running sessions for months really on Zoom, and we've been doing exercise, we've been cooking, we've done karaoke, we've done mindfulness. We have that in the morning before we start our day and it generally, it really helps. Also they've got a helpline as well. (Helen, S33—women with a learning disability and mental health issues).

The third sector not only acted to bring people together, they also provided direct services. Glasgow Disability Alliance for example, provided emergency support, preparing and delivering food to its members:

The people that are helping out, most, are the charities, they're catching people falling through the net. If it wasn't for, you know, you see online, like the Food Train, and they're delivering food to older people, or the Glasgow Disability Alliance are doing the same, and reaching out to people, then people would, you know, they would just be, I don't know where they would be, you know. (Caitlin, S07, living with complex health needs).

Third sector organisations have been flexible and changing the way they have worked, to help meet in the needs of those they work with. The approach contrasts markedly with that of the statutory agencies.

Whilst people have experienced real predicaments, hardships and uncertainties as a result of the withdrawal or reduction of their established social care, some people told us that this has been ameliorated by the exceptional support from individual health, social care and educational workers, as well as from the pivoting of the third sector. Without state investment, this support cannot be maintained, and any future withdrawal or reduction either in funding for the third sector or of social care will reinforce and amplify the harm already done by the pandemic.

### 3.3 Touch and presence

The pandemic has been a moment when social media and online communications have been more important than at perhaps any time in their history. Many areas of life—health, education, employment, retail, entertainment—have been largely and sometimes exclusively accessed via the Internet. This has benefitted many disabled people. Not only have they been able to be safe during the pandemic, but they have also avoided many of the barriers to which they are usually subjected. No matter if transport or buildings are physically inaccessible, or are tiring to use, if everyone is online. No matter if you find it hard to interact with people, if you can do so online and not even turn your webcam on.

All this presumes that people have access to the Internet. For those without tablets, laptops or other computer access, or Internet access, then there has been a double exclusion. Disabled adults make up a large proportion of non‐Internet users: in 2017, 56% of adult Internet non‐users were disabled, while only 22% of the UK population are disabled (Office for National Statistics, 2019a, 2019b). As well as this digital divide, others may have computer and Internet access, but lack privacy to learn or work or shop or interact with others as they would wish too. The best disability organisations understood the threat of a digital divide, and moved fast to prevent it. Glasgow Disability Alliance, for example, converted their budget for events into funding for electronic notebooks and online access, so that disabled people at risk of social exclusion could access the Internet:

They're also supplying me with a brand‐new iPad so that I can then take part in more Zoom events and training until the groups can actually meet again in person. So I must admit they have been a saving grace in all this… (Hannah, S21,).

Similarly, The Family Fund shifted its funding from providing holidays and short breaks to providing funding for computing, new technologies and Internet access. Entirely new networks, such as ‘The Staying Inn’ have been established for people with learning disabilities and organisations have used social media to bring disabled people together. There are of course concerns here: some worry that technology may be used to further exclude disabled people in the future. Ashley, who has multiple sclerosis, believes technology may limit efforts to include disabled people in other ways:

What worries me is that I don't want that, after this crisis is over, for people to say, oh, well actually we don't need to make that meeting accessible, because disabled people, you can Zoom it, or WebEx it, or Teams it. And that really worries me that you know, actually this whole online connectivity will lead to more isolation. Not less. Of course it is wonderful, for some people it is absolutely fantastic and brilliant, but I don't want it to be the only thing. (Ashley, E05).

Online access can do many things. But it cannot replace human touch and togetherness (Zulueta, 2020). Touch is central to the work of primary care doctors and health workers, rehabilitation therapists, and social care workers (Zulueta, 2020). Children crave to be in school with their peers. Adults want to go to work, or to day centres, to see their friends. This highlights the value of being together in real life—in both impairment‐specific and generic groups, and of human touch. Collective gatherings are necessary for almost everyone's sense of self and wellbeing. COVID has provided insight that technology is not an adequate substitute for real life. As one respondent said to us: ‘*You cannot play pool on Zoom’* (S34 Anne).

### 3.4 Messaging and leadership

Every single public announcement has been around keeping the vulnerable safe. You have to keep the vulnerable safe – that's been the main highlighted propaganda announcement from both…from Government at all levels. Yet what they actually do is to cut services to vulnerable people. (S02, Jonathan).

The fourth key message concerns how government has communicated about the pandemic. Clear communication in a health emergency is indispensable (Goggin & Ellis, 2020). But there has been frustration, verging on fury, particularly over the actions of the UK government. For example, text messages and letters about shielding were, initially at least, a failure. Shielding letters were received by a minority of our sample. Many that did not were then placed in a ‘responsibilisation’ predicament (Liebenberg et al., 2015); they needed to make the choice about whether and how to electively shield due to specific health concerns, but without the formal protections and benefits accrued through receiving a letter. Many people were also unsure about the steps they had to take to protect themselves: some went to extremes, while others choose to be less restrictive. Communication about the easing‐off of restrictions was also very badly handled and many people were unsure what they could and could not do, and what was safe for them and what was not.

Daily announcements of death tolls suggested that deaths of younger people were usually associated with ‘pre‐existing conditions’, as if this was somehow more acceptable. This not only sent a very negative message to disabled people, but it also made them very fearful, in many cases unnecessarily. As a result, we believe that some people have been shielding who did not need to be, from a health perspective. Kathleen, who has a functional movement disorder, highlighted this point:

You know, we've recently had people being told by text oh, you're no longer in the highly vulnerable group, and so I'm not quite sure where it screwed up there, but I actually am looking more at the consultants than the government on that one actually. They were supposed to have told their patients by letter why they weren't in the highly vulnerable group. Now, that might be the quick clinical decision. But I do wish those consultants had A, bothered to write the letter. But B, bothered to think it through. Because some of those people that were in that group what might not be highly vulnerable medically, and just vulnerable. (Kathleen, E21).

The science about COVID‐19 has been evolving, which cannot be blamed on anyone, but the messaging as to which people needed to shield and which did not have to continued to be unclear. Shielding places significant strain on health and wellbeing.

Government daily briefings were necessary to highlight the general public health significance of COVID‐19, particularly for those who struggle with online media, but were not sufficient to help people with learning difficulties in particular to understand what they should do differently. Nor has there been routine sign language interpretation of UK government briefings, which sends a very negative message to all disabled people in England. Also in contrast to the UK Government, the Scottish Government has held daily televised briefings throughout, benefitting Maurice who has learning disabilities:

The television is really important to Maurice, and I think that if it wasn't for the government spokespeople doing that regular briefing by television, he wouldn't have any meaningful information about what's going on … he doesn't understand a lot of what they're saying, but because there's a consistent format to it … he phones me every day after it to find out what was actually being said, and what it means for him…. (S25 interview with Maurice's guardian).

In this confusion, the third sector have also struggled to make sense of the Government's messaging and have often been left as much in the dark as the communities they serve. Voluntary organisations and schools have received conflicting information, often at the very last minute. Nevertheless, our evidence is that many disabled people's organisations and other community groups have played a key information role, getting the right message across. They have translated information into Easy Read and disseminated it via their webpages. Throughout this process they have continued to be trusted, whereas government (at least in England) rapidly lost that trust. The position in Scotland has been slightly different, and there has been more trust in the Scottish Government:

Nicola Sturgeon has been very clear, concise, empathetic. It doesn't help you, but it's all been very clear and direct. (Ingrid, S13).

However, it has been unclear to some people whether to follow instructions from London or Edinburgh.

Positive messaging was undermined by a few well‐publicised errors of judgement—such as blanket use of Do Not Attempt Resuscitation without discussion in one instance, and the National Institute of Health and Care Excellence equation of social care with extreme dependency—at the start of the pandemic, which led to a suspicion amongst some disabled people that they would not receive fair treatment, particularly among those with complex health conditions:

We honestly felt, if we'd went into hospital, we would have been denied a ventilator because of our conditions. And we'd rather have died here together, than be taken in. (Caitlin, S07).

## 4 DISCUSSION AND CONCLUSIONS

The COVID‐19 pandemic has been a terrible time for all those who have lost loved ones, and for NHS who have borne the brunt of caring for people affected by the virus. But it has also exposed inequalities and differing vulnerabilities within British society that may have previously been obscured or ignored (Bambra et al., 2020; Scambler, 2020). Not only have many disabled people been at greater risk of both contracting the virus—because of reliance on social care, for example—but some people who are older or have co‐morbidities have been at greater risk of dying or having severe adverse consequences.

How relevant is this paper to wider policy? We researched only in England and Scotland, but Northern Ireland has a similar social care system to Scotland. Wales is different again from England and Scotland. We did not recruit enough people from BME backgrounds to say anything about the specifics of that experience. We cannot say much about care homes, because we only included seven people who lived in residential settings, and few older disabled people. However, we spoke to infrastructure organisations who could speak about these experiences. Social care was not working as it should be prior to the pandemic—and has been exposed even more during it, as Daly (2020) explains in her analysis of failure to manage the COVID‐19 impact in UK care homes. Our data confirms this, and the reversion to family as carers of last resort.

Most respondents in this study had Internet access, although we did make efforts to speak to some individuals who did not. It should be remembered that although we have spoken to many people, achieving both breadth and depth, this is a qualitative study, so it cannot be representative in the same way as a survey. We should also note how Internet interviews overcame barriers of remoteness and distance in many cases, facilitating data collection.

Our data suggest that many disabled people and their families have felt abandoned and forgotten during the pandemic (Simmons, 2020). Bambra et al. (2020) have drawn on ideas developed by Singer on the impact of HIV and AIDS to discuss COVID‐19 as a syndemic, where a pandemic becomes magnified by pre‐existing inequalities and co‐morbidities. According to Bambra et al. (2020) COVID‐19 has acted as ‘co‐occurring, synergistic pandemic that interacts with and exacerbates’ pre‐existing conditions, social inequalities and disadvantage and poverty. For disabled people it has exposed and magnified existing structural failings and inequalities and has differentially impacted on disabled people; in many cases their needs were not protected and the response of the state has compromised their human rights.

Policies need to be put in place to try and ameliorate and rectify the harm caused to disabled people by the pandemic. Lessons need to be learned from the difficulties and solutions identified in the pandemic, both to enable better provision in any subsequent wave of COVID‐19 or future pandemics. This information also needs to be shared to nform responses in other countries (Kuper et al., 2020). Data is already emerging to suggest that, as in the UK, disabled people have felt the brunt of the pandemic internationally (Inclusion Europe, 2020).

### 4.1 As a matter of urgency

The needs of disabled people have to be fully considered in COVID responses. Assessments need to consider the implications of decisions on different impairment groups and those with combined impairments. Thought also needs to be given to how decisions are communicated. Easy Read formats need to be easily available for all key decisions: whereas organisations were funded to provide this, people with learning disabilities did not know where to go to access this. Sign language interpretation should be provided for daily government broadcasts, because it is practically but also symbolically important. These serve two functions, they not only directly inform groups of disabled people, they also serve a cultural purpose, making disabled people feel included.

At a minimum and as a matter of urgency, local authorities should make it clear that social care packages will be fully reinstated and resources will be invested to address the backlog in social care assessments. Prior to the pandemic many of these services had already been cut and many disabled people already felt that their needs were not being fully met (Glasby, 2020) COVID‐19 has magnified many of the pre‐existing problems with social care (Bottery, 2020). Some research participants expressed concern that their social care would not be reinstated to their pre‐COVID level, justified by the fact that they had survived during the pandemic. Given the prospective financial position of local authorities, this is a legitimate fear. Social care provision is central to enabling disabled people to live independently in society and a reversion to the family as social care of last resort is not acceptable.

The reestablishment of social supports and services, including day centres and other activities, is urgently needed to support disabled people, particularly people with learning disabilities and people with autism. COVID‐19‐safe alternatives need to be developed and health and social care funders and providers must to work with disabled people and their organisations to develop new ways of delivering support. The withdrawal of these services has had a detrimental impact on these individuals and put intense pressure on their families and other support networks.

Measures need to be taken to ensure that disabled children receive support to ‘catch up’ on the educational provision that they were excluded from during the pandemic. Again, here there are many pre‐existing problems faced by children with special educational needs (Chatzitheochari et al., 2016). They are more likely to live in poverty and less likely to have access to new technologies (Black, 2019), both of which have been linked to less intensive home learning during the pandemic (Cullinane & Montacute, 2020).

Health and rehabilitation services need to urgently address the physical health needs of disabled people; this includes impairment and non‐impairment related needs. Services such as physiotherapy and speech and language therapy have to urgently undertake needs assessments and develop plans to help to tackle the problems faced by disabled people. This is particularly true for habilitation for disabled children and young people. The provision, repair and service of assistive products and aids to daily living needs to be prioritised.

The third sector played a central role in supporting disabled people through the pandemic, with both practical and social support. Third sector organisations were essential to maintaining services; their actions were swift and decisive and without them things would have been much, much worse. For some, the sector was a life saver. The precarity of the third sector needs to be addressed ensuring it can continue to provide support to disabled people and their families. Three ways to achieve this are: to work with the sector as equal partners rather than contractors; to reduce unnecessary reporting and administration; and to provide fair and longer‐term funding.

### 4.2 In the medium to long term

The social care system has been broken for some time; its vulnerability has been exposed by the pandemic. The current system is not working for disabled people nor is it working for those employed to provide social care (Glasby et al., 2020). Years of austerity measures and cuts to social care harmed our ability to respond to this pandemic. An overhaul of the system is required that places the individual and their care at the centre. A system is needed that is responsive and humane. In order to achieve good quality social care provision, secure funding is required.

Policymakers and social care providers must work collaboratively with disabled people and their organisations to address their needs during the rest of this pandemic and after and in anticipation of comparable future crises. The pandemic has significantly impacted on the health and wellbeing of disabled people and Governments in Edinburgh and London must put in place systems to measure the challenges disabled people face and develop strategies and polices to help reduce their impact in the future. Post‐pandemic social change is required to enable disabled people not only to regain what has been lost through the pandemic, but also to gain full citizenship rights in the United Kingdom.

## CONFLICT OF INTEREST

The authors have no conflict of interest to declare.

## ETHICS STATEMENT

This research was given ethical approval by the London School of Hygiene and Tropical Medicine Research Ethics Committee.

## Data Availability

Research data are not shared.

## References

[spol12758-bib-0001] Andrew, A. , Cattan, S. , Costa Dias, M. , Farquharson, C. , Kraftman, L. , Krutikova, S. , … Sevilla, A. (2020). Inequalities in Children's experiences of home learning during the COVID‐19 lockdown in England. Fiscal Studies, 41(3), 653–683. 33362314 10.1111/1475-5890.12240 PMC7753283

[spol12758-bib-0002] Bambra C. , Riordan R. , Ford J. , & Matthews F. (2020). The COVID‐19 pandemic and health inequalities. Journal of Epidemiology and Community Health, 74(11), 964–968. 10.1136/jech-2020-214401. 32535550 PMC7298201

[spol12758-bib-0003] Bibby, J. , Everest, G. , & Abbs, I. (2020). Will COVID‐19 be a watershed moment for health inequalities? The Health Foundation. 1–20. https://www.health.org.uk/sites/default/files/2020-05/Will%20COVID-19%20be%20a%20watershed%20moment%20for%20health%20inequalities.pdf (Consulted January 24, 2021).

[spol12758-bib-0004] Black, A. (2019). A picture of special educational needs in England—An overview. Frontiers in Education, 4, 79. 10.3389/feduc.2019.00079

[spol12758-bib-0005] Bottery, S. 2020. How Covid‐19 has magnified some of social care's key problems. https://www.kingsfund.org.uk/publications/covid-19-magnified-social-care-problems (Consulted January 26, 2021)

[spol12758-bib-0006] Campbell, J. 2020. “Meet the vulnerables”, transcript of BBC Radio 4 programme. https://www.bbc.co.uk/news/disability-52713752 (Consulted January 6th 2021).

[spol12758-bib-0007] Chatzitheochari, S. , Parsons, S. , & Platt, L. (2016). Doubly disadvantaged? Bullying experiences among disabled children and young people in England. Sociology, 50(4), 695–713. 27546915 10.1177/0038038515574813 PMC4963628

[spol12758-bib-0008] Cullinane, C. , & Montacute, R. (2020). COVID‐19 and social mobility impact brief# 1: School shutdown. London: The Sutton Trust.

[spol12758-bib-0009] Daly, M. (2020). COVID‐19 and care homes in England: What happened and why? Social Policy and Administration, 54(7), 985–998. 10.1111/spol.12645 PMC7461496 32904948

[spol12758-bib-0010] Deakin, H. , & Wakefield, K. (2013). Skype interviewing: Reflections of two PhD researchers. Qualitative Research, 14(5), 603–616. 10.1177/1468794113488126

[spol12758-bib-0011] Dickinson, H. , Carey, G. , & Kavanagh, A. M. (2020). Personalisation and pandemic: An unforeseen collision course? Disability and Society, 35(6), 1012–1017. 10.1080/09687599.2020.1772201

[spol12758-bib-0012] Glasby, J. , Zhang, Y. , Bennet, M. R. , & Hall, P. (2020). A lost decade? A renewed case for adult social care reform in England. Journal of Social Policy, 1–32. 10.1017/S0047279420000288

[spol12758-bib-0046] Glasby, J. , Zhang, Y. , Bennett, M. R. , & Hall, P. (2021). A lost decade? A renewed case for adult social care reform in England. Journal of Social Policy, 50(2), 406–437. 10.1017/s0047279420000288

[spol12758-bib-0013] Glasgow Disability Alliance . (2020). Supercharged: A Human Catastrophe Glasgow. Glasgow: Glasgow Disability Alliance. https://gda.scot/wp-content/uploads/2020/08/GDA–Supercharged-Covid-19Report.pdf

[spol12758-bib-0014] Glynn, J. R. , Fielding, K. , & Shakespeare, T. (2020). COVID‐19: Excess all cause mortality in domiciliary care. British Medical Journal, 370, m2751. 10.1136/bmj.m2751 32660936

[spol12758-bib-0015] Goggin, G. , & Ellis, K. (2020). Disability, communication, and life itself in the COVID‐19 pandemic. Health Sociology Review, 29(2), 168–176. 10.1080/14461242.2020.1784020 33411654

[spol12758-bib-0016] Greater Manchester Coalition of Disabled People . (2020). Greater Manchester disabled people's panel: GM big disability survey: Covid‐19. Manchester: Greater Manchester Coalition of Disabled People.

[spol12758-bib-0017] Guest, G. , MacQueen, K. , & Namey, E. (2012). Applied thematic analysis. London: Sage.

[spol12758-bib-0018] Harrison, S. L. , Fazio‐Eynullayeva, E. , Lane, D. A. , Underhill, P. , & Lip, G. Y. H. (2020). Comorbidities associated with mortality in 31,461 adults with COVID‐19 in the United States: A federated electronic medical record analysis. PLoS Medicine, 17(9), e1003321. 10.1371/journal.pmed.1003321 32911500 PMC7482833

[spol12758-bib-0019] Hupkau, C. , & Petrongolo, B. (2020). Work, care and gender during the COVID‐19 crisis. Fiscal Studies, 41(3), 623–653. 33362313 10.1111/1475-5890.12245 PMC7753653

[spol12758-bib-0020] Inclusion Europe . (2020). Neglect and discrimination multiplied how Covid‐19 affected the rights of people with intellectual disabilities and their families. Brussels: Inclusion Europe. https://www.inclusion-europe.eu/wp-content/uploads/2020/11/COVID-report-Final.pdf

[spol12758-bib-0021] Inclusion London . (2020). Abandoned, forgotten, ignored: The impact of the coronavirus pandemic on disabled people: Interim report. London: Inclusion London. https://www.inclusionlondon.org.uk/wp-content/uploads/2020/06/Abandoned-Forgotten-and-Ignored-Final-1.pdf

[spol12758-bib-0022] Inclusion Scotland . (2020). Rights at risk: Covid‐19, disabled people and emergency planning in Scotland. Edinburgh: Inclusion Scotland.

[spol12758-bib-0023] Institute for Government . 2019. Performance tracker: Adult social care https://www.instituteforgovernment.org.uk/publication/performance-tracker-2019/adult-social-care

[spol12758-bib-0024] Johnson, P. , Joyce, R. , & Platt, L. (2021). The IFS Deaton review: A new year message. London Institute for Fiscal Studies. https://ifs.org.uk/uploads/IFS‐Deaton‐Review‐New‐Year‐Message.pdf

[spol12758-bib-0025] Kuper H. , Banks L. M. , Bright T. , Davey C. , & Shakespeare T. (2020). Disability‐inclusive COVID‐19 response: What it is, why it is important and what we can learn from the United Kingdom’s response. Wellcome Open Research, 5, 79. 10.12688/wellcomeopenres.15833.1. 32500099 PMC7236579

[spol12758-bib-0026] Learning Disabilities Mortality Review (LeDeR) Programme . (2020). Deaths of people with learning disabilities from COVID‐19. Bristol: University of Bristol.

[spol12758-bib-0027] Liebenberg, L. , Ungar, M. , & Ikeda, J. (2015). Neo‐liberalism and responsibilisation in the discourse of social service workers. The British Journal of Social Work, 45(3), 1006–1021.

[spol12758-bib-0028] Lobe, B. , Morgan, D. , & Hoffman, K. A. (2020). Qualitative data collection in an era of social distancing. International Journal of Qualitative Methods, 19 1609406920937875, 1–18.

[spol12758-bib-0029] Mason, D. M. , & Ide, B. (2014). Adapting qualitative research strategies to technology savvy adolescents. Nurse Researcher, 21(5), 40–45. 10.7748/nr.21.5.40.e1241 24877910

[spol12758-bib-0030] Mealer, M. , & Jones, J. (2014). Methodological and ethical issues related to qualitative telephone interviews on sensitive topics. Nurse Researcher, 21(4), 32–37. 10.7748/nr2014.03.21.4.32.e1229 24673351

[spol12758-bib-0031] Mladenov, T. (2015). Neoliberalism, postsocialism, disability. Disability and Society, 30(3), 445–459.

[spol12758-bib-0032] Morris, J. (2011). Rethinking disability policy. York: Joseph Rowntree Foundation. https://www.jrf.org.uk/sites/default/files/jrf/migrated/files/disability-policy-equality-summary.pdf

[spol12758-bib-0033] Office for National Statistics . (2019a). Improving disability data in the UK. London: ONS.

[spol12758-bib-0034] Office for National Statistics . (2019b). Exploring the UK's digital divide. London: ONS. https://www.ons.gov.uk/peoplepopulationandcommunity/householdcharacteristics/homeinternetandsocialmediausage/articles/exploringtheuksdigitaldivide/2019‐03‐04#:~:text=For%20internet%20non‐users%20aged,aged%2075%20years%20and%20older

[spol12758-bib-0035] Office for National Statistics . (2020a). Coronavirus (COVID‐19) related deaths by disability status, England and Wales: 2 March to May 15, 2020. Deaths related to the coronavirus (COVID‐19) by disability status, including death counts, age‐standardised mortality rates, and hazard rate ratios by age, sex and disability status. London: ONS.

[spol12758-bib-0036] Office for National Statistics . (2020b). Coronavirus and depression in adults, Great Britain: June 2020. The proportion of the population with depressive symptoms in Great Britain between 4 and June 14, 2020, based on the Opinions and Lifestyle Survey. Includes how symptoms of depression have changed since before the pandemic (July 2019 to March 2020). London: ONS.

[spol12758-bib-0037] Office for National Statistics . (2020c). Coronavirus and the social impacts on disabled people in Great Britain: September 2020. London: ONS.

[spol12758-bib-0038] Public Health England . (2020). Disparities in the risk and outcomes of COVID‐19. London: PHE. https://assets.publishing.service.gov.uk/government/uploads/system/uploads/attachment_data/file/908434/Disparities_in_the_risk_and_outcomes_of_COVID_August_2020_update.pdf

[spol12758-bib-0039] Scambler, G. (2020). Covid‐19 as a ‘breaching experiment’: Exposing the fractured society. Health Sociology Review, 29(2), 140–148. 10.1080/14461242.2020.1784019 33411650

[spol12758-bib-0040] Simmons, A. L. (2020). COVID‐19 social distancing: A snippet view of the autistic social world. Disability and Society, 35(6), 1007–1011. 10.1080/09687599.2020.1774866

[spol12758-bib-0041] Sinnathamby, M. A. , Whitaker, H. , Coughlan, L. , Bernal, J. L. , Ramsay, M. , & Andrews, N. (2020). All‐cause excess mortality observed by age group and regions in the first wave of the COVID‐19 pandemic in England. Eurosurveillance, 25(28), 2001239. 10.2807/1560-7917.ES.2020.25.28.2001239 PMC7376843 32700669

[spol12758-bib-0042] Theis N. , Campbell N. , De Leeuw J. , Owen M. , & Schenke K. C. (2021). The effects of COVID‐19 restrictions on physical activity and mental health of children and young adults with physical and/or intellectual disabilities. Disability and Health Journal, 14(3), 101064. 10.1016/j.dhjo.2021.101064. 33549499 PMC7825978

[spol12758-bib-0043] World Health Organization . (2011). World report on disability. Geneva: WHO.

[spol12758-bib-0044] Yang, J. , Zheng, Y. , Gou, X. , Pu, K. , Chen, Z. , Guo, Q. , … Zhou, Y. (2020). Prevalence of comorbidities and its effects in patients infected with SARS‐CoV‐2: A systematic review and meta‐analysis. International Journal of Infectious Diseases, 94, 91–95. 32173574 10.1016/j.ijid.2020.03.017 PMC7194638

[spol12758-bib-0045] Zulueta, P .de. 2020. Touch matters: COVID‐19, physical examination and 21st century general practice. British Journal of General Practice, 70 (701): 594–595. DOI: 10.3399/bjgp20X713705 PMC7707044 33243905

